# Molecular Profiles of Advanced Urological Cancers in the PERMED-01 Precision Medicine Clinical Trial

**DOI:** 10.3390/cancers14092275

**Published:** 2022-05-03

**Authors:** Emilien Billon, Gwenaelle Gravis, Arnaud Guille, Nadine Carbuccia, Jose Adelaide, Séverine Garnier, Pascal Finetti, Emilie Denicolaï, Patrick Sfumato, Serge Brunelle, Jeanne Thomassin-Piana, Géraldine Pignot, Jochen Walz, Christian Chabannon, Jihane Pakradouni, Renaud Sabatier, Cécile Vicier, Cornel Popovici, Emilie Mamessier, Anthony Gonçalves, Daniel Birnbaum, Max Chaffanet, François Bertucci

**Affiliations:** 1 Laboratory of Predictive Oncology, Centre de Recherche en Cancérologie de Marseille (CRCM), Institut Paoli-Calmettes, INSERM UMR1068, CNRS UMR725, Aix-Marseille University, 13009 Marseille, France; billone@ipc.unicancer.fr (E.B.); guillea@ipc.unicancer.fr (A.G.); carbuccian@ipc.unicancer.fr (N.C.); adelaidej@ipc.unicancer.fr (J.A.); garniers@ipc.unicancer.fr (S.G.); finettip@ipc.unicancer.fr (P.F.); denicolaie@ipc.unicancer.fr (E.D.); sabatierr@ipc.unicancer.fr (R.S.); vicierc@ipc.unicancer.fr (C.V.); emilie.mamessier@inserm.fr (E.M.); goncalvesa@ipc.unicancer.fr (A.G.); birnbaumd@ipc.unicancer.fr (D.B.); chaffanetm@ipc.unicancer.fr (M.C.); 2 Department of Medical Oncology, Institut Paoli-Calmettes, 13009 Marseille, France; gravisg@ipc.unicancer.fr; 3 Biostatistics Unit, Institut Paoli-Calmettes, 13009 Marseille, France; sfumatop@ipc.unicancer.fr; 4 Department of Imaging, Institut Paoli-Calmettes, 13009 Marseille, France; brunelles@ipc.unicancer.fr; 5 Department of Biopathology, Institut Paoli-Calmettes, 13009 Marseille, France; thomassinj@ipc.unicancer.fr; 6 Department of Urology, Institut Paoli-Calmettes, 13009 Marseille, France; pignotg@ipc.unicancer.fr (G.P.); walzj@ipc.unicancer.fr (J.W.); 7 Biobank, Department of Hematology, Institut Paoli-Calmettes, 13009 Marseille, France; chabannonc@ipc.unicancer.fr; 8 Department of Clinical Research and Innovation, Institut Paoli-Calmettes, 13009 Marseille, France; pakradounij@ipc.unicancer.fr; 9 Department of Oncogenetics, Institut Paoli-Calmettes, 13009 Marseille, France; popovicic@ipc.unicancer.fr

**Keywords:** advanced urological cancers, mutation, PERMED-01 trial, precision medicine, sequencing, array-CGH, t-NGS

## Abstract

**Simple Summary:**

The goal of precision medicine is to deliver therapy matched to a relevant actionable genetic alteration (AGA) identified in the tumor. Few data are available regarding precision medicine in advanced urological cancers (AUC), the prognosis of which remains unfavorable. Sixty-four patients with refractory AUC were enrolled in the PERMED-01 clinical trial and underwent a tumor biopsy that was then profiled using sophisticated molecular analyses. The results were discussed in real-time during a weekly molecular tumor board meeting, and patients with a relevant AGA became candidates for an eventual matched therapy. A complete molecular profile was obtained in 77% of cases and an AGA was identified in 59%. Nineteen percent of patients received a matched therapy on progression, of which 42% showed a clinical benefit. The objective response, disease control rates, and the 6-year overall survival were higher in the “matched therapy group” than in the “non-matched therapy group”.

**Abstract:**

Introduction. The prognosis of advanced urological cancers (AUC) remains unfavorable, and few data are available regarding precision medicine. Methods: the PERMED-01 prospective clinical trial assessed the impact of molecular profiling in adults with refractory advanced solid cancer, in terms of number of patients with tumor actionable genetic alterations (AGA), feasibility, description of molecular alterations, treatment, and clinical outcome. We present here those results in the 64 patients enrolled with AUC. DNA extracted from a new tumor biopsy was profiled in real-time (targeted NGS, whole-genome array-comparative genomic hybridization), and the results were discussed during a weekly molecular tumor board meeting. Results: a complete molecular profile was obtained in 49 patients (77%). Thirty-eight (59%) had at least one AGA. Twelve (19%) received a matched therapy on progression, of which 42% had a PFS2/PFS1 ratio ≥ 1.3 versus 5% in the “non-matched therapy group” (*n* = 25). The objective response and disease control rates were higher in the “matched therapy group” (33% and 58%, respectively) than in the “non-matched therapy group” (13% and 22%), as was the 6-month OS (75% vs. 42%). Conclusion: the profiling of a newly biopsied tumor sample identified AGA in 59% of patients with AUC, led to “matched therapy” in 19%, and provided clinical benefit in 8%.

## 1. Introduction

Urological cancers represent ~2 million cases diagnosed worldwide [1]. Better understanding of oncogenesis led to the development of new therapies. They include tyrosine kinase inhibitors, mTOR inhibitors, and anti-VEGF/VEGFR targeting therapy in clear cell renal cell carcinoma, and more recently immune checkpoint inhibitors [2]. Androgen-deprivation therapy remains the basis of treatment for advanced prostate cancer (PC) and new-generation hormone therapy (abiraterone, enzalutamide, apalutamide) recently improved the management [3]. Immune therapy and targeted therapy against nectin-4 and FGFR were recently approved in advanced urothelial cancers [4,5]. Despite these progresses, the prognosis of advanced urological cancers (AUC) remains poor. In all cases, the therapeutic choice remains based on pathological analysis.

High-throughput molecular profiling drew the omic landscape of urological tumors [6,7,8], evidencing in each cancer type few relatively frequent drivers, and many rarer drivers shared with other cancers and potentially actionable. These latter provide opportunities for therapeutic targeting. The first clinical trials of precision medicine showed feasibility and interesting results in trials, including diverse tumor types [9,10,11] or dedicated to a specific tumor type, such as lung or breast cancer [12,13]. Data are rarer regarding AUC [5,14,15].

PERMED-01 was a prospective clinical trial of precision medicine dedicated to patients with refractory advanced solid cancers. Its principal objective was to determine the number of patients with actionable genetic alterations (AGA) in tumor samples [16]. A secondary objective was to describe the results per cancer type. This is our present objective in patients with AUC.

## 2. Materials and Methods

### 2.1. Study Design

The PERMED-01 prospective clinical trial was conducted in our institution (Paoli-Calmettes Institute, Marseille, France). It was dedicated to patients with refractory advanced solid cancer and included a mandatory tumor biopsy for real-time molecular profiling. The profiling included targeted next-generation sequencing (t-NGS) and array-comparative genomic hybridization (aCGH). The trial was approved by our Institutional Review Board, a national ethics committee (CPP Sud-Méditerranée), and the French National Agency for Medicines and Health Products Safety. It is registered as ClinicalTrials.gov identifier NCT02342158, and was conducted in accordance with the Good Clinical Practice guidelines of the International Conference on Harmonization. The patients’ informed consent was mandatory before inclusion.

### 2.2. Inclusion Criteria

Inclusion criteria were as follows: age ≥ 18 years, pathologically diagnosed solid cancer, of locally advanced or metastatic stage, with progression after at least one line of prior systemic therapy and one lesion accessible for biopsy. Other criteria included an Eastern Cooperative Oncology Group (ECOG) Performance Status ≤ 2, affiliation to Social Insurance, and signed informed patient’s consent. The main exclusion criteria were bone or brain metastasis as sole metastatic site, and symptomatic or progressive nervous central system metastases. Five hundred and fifty patients were enrolled in the trial over the inclusion period [16]. The present study is limited to the subpopulation of patients with urological cancer (prostate, kidney, bladder/ureter, and testicular cancers).

### 2.3. Genome Analysis

After the patient’s enrollment, a tumor biopsy or resection was planned, and the collected sample was frozen and submitted for molecular analysis. Only samples displaying a tumor cellularity superior or equal to 30% of tumor cells were retained for tumor DNA extraction and aCGH and t-NGS. Regarding t-NGS, we used home-made panels of genes previously selected for their involvement in cancers: three chronologically extended panels (Table S1) covering 395, 494, and 560 genes were tested, respectively, and included from 49 to 66 cancer predisposition genes from the BROCA Cancer Risk panel (https://testguide.labmed.uw.edu/public/view/BROCA, accessed on 18 December 2013). A total of 200 ng of tumor samples and matched normal blood samples (available for 26 patients) were sequenced. The respective median depths were 743x and 396x. The alignment of sequence data to the human genome (UCSC hg19) was done as described [17], as well as variants calling and annotation [18]. In the 26 patients with a matched normal sample sequenced, the Tumor mutational burden (TMB) and MSI-H (MicroSatellite Instability-High) status were defined. The cut-off for TMB-high was 10 mutations/Mb. Microsatellite instability detection was done using the software MSIsensor [19], which computes a “MSI score” and a 10% cut-off to detect MSI-H tumors. MSIsensor is an efficient and effective tool for deriving MSI status from standard tumor-normal paired sequence data. This C++ program computes length distributions of microsatellites per site in paired tumor and normal sequence data, subsequently using these to statistically compare observed distributions in both samples. Array-CGH experiments were done using high-resolution microarrays (SurePrint G3 Human CGH Microarray Kit, Agilent Technologies, Massy, France). All probes were mapped according to the hg19/NCBI human genome mapping database. A total of 500 ng of tumor DNA was used. We limited analysis to the 565 genes present in at least one used NGS panel. The DNA copy-number alterations were defined as follows: amplification (Log2ratio > 1) or deletion (Log2ratio < −1). A HRD (Homologous Recombination Deficiency) score, based on losses of heterozygosity (LOH), was calculated for each tumor sample from all tested aCGH genes [20]: a score ≥ 10 was considered as HRD-high. The generated molecular data have been deposited in the following public databases: European Genome-phenome Archive (EGA: accession EGAS00001004554) for NGS and EMBL-EBI (E-MTAB-9998 accession number) for aCGH.

### 2.4. Multidisciplinary Molecular Tumor Board

For each patient, two scientists and one bioinformatician reviewed all molecular profiles and generated the molecular report. This latter was then presented and discussed during our weekly institutional molecular tumor board (MTB) meeting in order to recommend and prioritize an eventual AGA-matched therapy. Actionability of molecular alterations was defined in real-time by the existence of a therapy targeting the altered protein, either directly or indirectly by impacting the activated pathway. For oncogenes, we retained focal gene amplifications (≥6 copies), hotspot mutations activating or associated with therapeutic resistance, and mutations with undescribed pathogenic effect but with characteristics suggesting a pathogenic effect (kinase domain or other critical protein domain). For tumor suppressor genes, we considered homozygous deletion, heterozygous deletion associated with a loss of expression (IHC: immunohistochemistry), heterozygous deletion associated with a known inactivating mutation, and heterozygous deletion associated with a mutation with undescribed pathogenic effect but with characteristics suggesting a pathogenic effect. The evidence level of the biomarker/treatment association was estimated by using OncoKB (from 1 to 4) [21] and clinical or pre-clinical data from the literature, suggesting an association with response or resistance and/or the existence of a clinical trial in which the alteration was mandatory for enrollment. AGAs corresponded not only to single-gene alterations, but also to genomic scores (high HRD, high TMB, and MSI-H). We considered the recommended therapy as matched when the recommendation was based upon the PERMED-01 molecular screening. Otherwise, it was defined as a non-matched therapy. Treatment delivery was at the discretion of physician and patient. Once treated, the patients were monitored for tumor response according to our institutional guidelines.

### 2.5. Objectives, Endpoints and Statistical Analysis

Our primary objective was to measure the number of patients for whom identification of AGAs in tumor samples could lead to the delivery of a matched therapy. Patient and disease characteristics were summarized by using counts and frequencies for categorical variables and medians (ranges) for continuous variables. Differences among the groups were assessed using the Fisher’s exact test or Mann–Whitney test, when appropriate. Secondary objectives were to report all somatic and germline molecular alterations identified, and to describe and compare, among the patients who received a systemic therapy for progression after PËRMED-01 enrollment (therapy 2), the group of patients with AGA who received a matched therapy versus the group of patients who received a non-matched therapy; such comparison concerned the clinicopathological characteristics including therapeutic efficacy. As the main efficacy endpoint, we used the ratio of progression-free survival (PFS2) on therapy 2 to the PFS on the immediate previous treatment (PFS1, therapy 1) [10]. This ratio, thereafter designated PFS2/PFS1 ratio, ≥1.3 is considered in the literature as a non-ambiguous sign of activity for the new treatment, relative to the previously received treatment [10]. The PFS was measured from the time of treatment start until progression for PFS1 and until progression or death for PFS2. Event-free patients were censored at the date of last contact. Univariate analysis searched for clinical variables associated with a ratio ≥ 1.3 (Fisher’s exact test). The tumor response was considered as assessable when the patient had received at least 8 weeks of treatment and was radiologically assessed according to Response Evaluation Criteria In Solid Tumors (RECIST version 1.1). Overall survival (OS) was measured from the start of therapy 2 until death or date of last news. Probabilities of PFS and OS were estimated using the Kaplan-Meier method and univariate associations were evaluated with the log-rank test. Statistical analyses were done either with the R software version 2.15 or Prism software (Graphpad software, San Diego, CA, USA) and the significance level was set to 5%. All statistical tests were two-sided.

## 3. Results

### 3.1. Patients’ Characteristics

**Figure 1:**
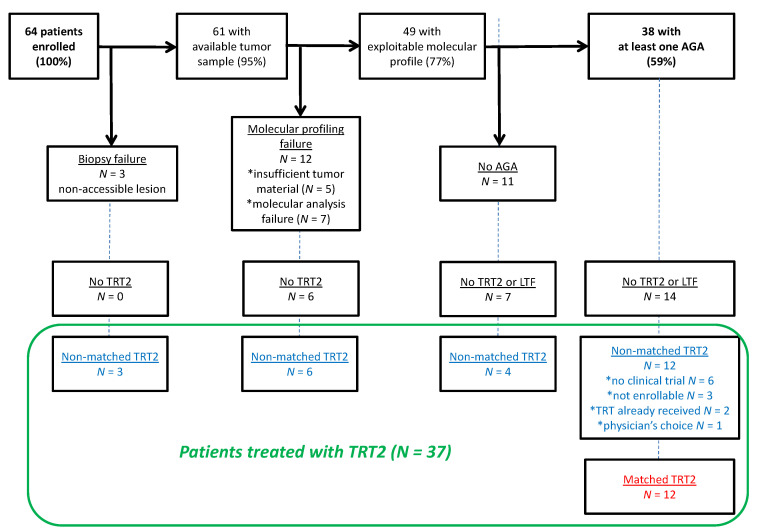
CONSORT diagram. TRT2, systemic treatment delivered for disease progression after PERMED-01 enrollment; AGA, actionable genetic alteration; LTF, lost to follow-up.

From December 2014 to October 2017, 64 patients with AUC were enrolled in PERMED-01 (12% of the cohort). Prostate cancer was the most frequent cancer (*N =* 39, 61%; Table 1), followed by bladder/ureter urothelial (*N =* 12, 19%), kidney (*N =* 9, 14%), and testis (*N =* 4, 6%) cancers. Tumor samples were obtained from 61 patients (95%) after biopsy (Figure 1) of a metastatic site in 51 patients, mainly liver (*N =* 22, 36%) and lymph nodes (*N =* 13, 21%), or of the primary tumor (16%) in 10 patients with PC. The advanced disease was metastatic in all but one patient. The median numbers of different metastatic sites and prior lines of systemic therapy for advanced disease were 2 (range, 0–7) and 3.5 (range, 1–11), respectively. One out of 64 patients experienced a grade ≥ 2 adverse event: grade 3 fever after prostate biopsy that completely recovered after antibiotic treatment.

### 3.2. Landscape of Somatic and Germline Molecular Alterations

**Figure 2:**
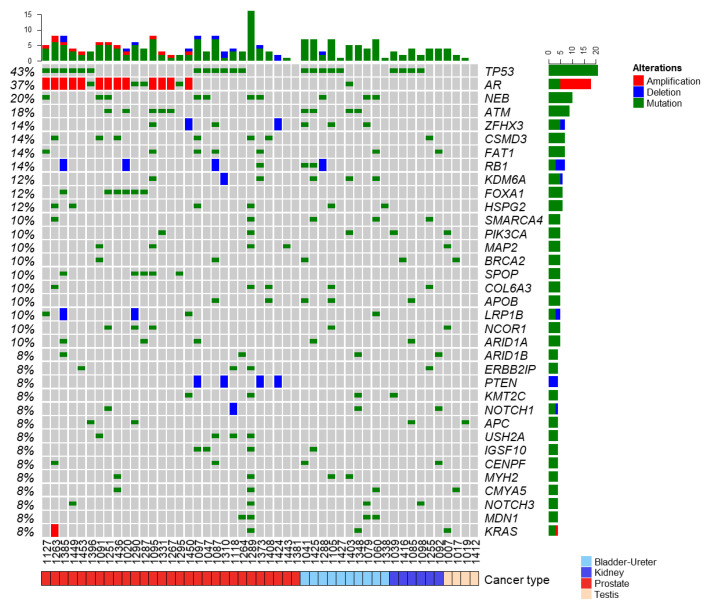
Repertoire of somatic alterations. Oncoprint of the top 35 genes altered in at least four out of 49 samples with exploitable molecular profile. Somatic alterations (mutations and can) color-coded according to the legend. The genes are ordered from top to bottom by decreasing percentage of altered tumors (right panel), and the tumors are ordered from left to right by cancer type then by the “memo sort” method, which can visualize the mutual exclusivity across genes. Bar charts (top) indicate the number of mutations for each sample. Bar charts (right) indicate the number of samples altered for each gene. The cancer type is shown at the bottom and is color-coded according to the legend.

Array-based CGH and t-NGS profiles were established from 49 patients (77%). The median time between inclusion and discussion during the MTB was 53 days (range 4–162). We found 633 somatic gene alterations, including 530 mutations and 103 copy number alterations (CNAs: 52 deletions, 51 amplifications). The 35 most frequently altered genes (Figure 2) notably included TP53 (43% of samples), AR (37%), NEB (20%), ATM (18%), FAT1, RB1, CSMD3, and ZFHX3 (14%), KDM6A, FOXA1, and HSPG2 (12%). Of note, some altered genes (FOXA1, SPOP, PTEN, and USH2A) were altered only in PC, in which (Figure S1) the most frequent alteration was AR amplification, followed by TP53 mutation; five genes involved in chromatin remodeling and transcriptional regulation (FOXA1, KDM6A, NCOR1, ARID1A, ZFHX3) were among the top 20 genes altered. Ten out of 29 PC patients (34%) showed an alteration in at least one of 15 genes tested in the PROfound trial [14], mainly ATM (*N =* 5) and BRCA2 (*N =* 2). A high HRD score was observed in 20 patients (41% of cases), including 13 PCs, 5 urothelial bladder cancers, 1 kidney cancer, and 1 testis cancer. A positive correlation existed between the HRD score and the presence/absence of mono-/bi-allelic pathogenic alterations of genes involved in homologous recombination (Figure S2). Two PC patients displayed focal tandem duplication associated with CDK12 bi-allelic loss. As expected, they did not show a high HRD score [22]. High TMB was observed in only 1/26 of informative patients (bladder cancer).

For 26 patients, the sequencing of germline DNA was done. Analysis limited to the predisposition genes of the BROCA Cancer Risk panel identified 48 germline variants (GVs) in 24 genes: four were pathogenic or likely pathogenic (PGVs) (Table S2) and 45 were variants of unknown significance (VUS). The PGVs, observed in four patients (15%, 95%CI 5–36), targeted genes related to DNA repair (ATM, BRCA2, CHEK2, and NBN).

### 3.3. Actionable Genetic Alterations and Matched Therapy

**Figure 3:**
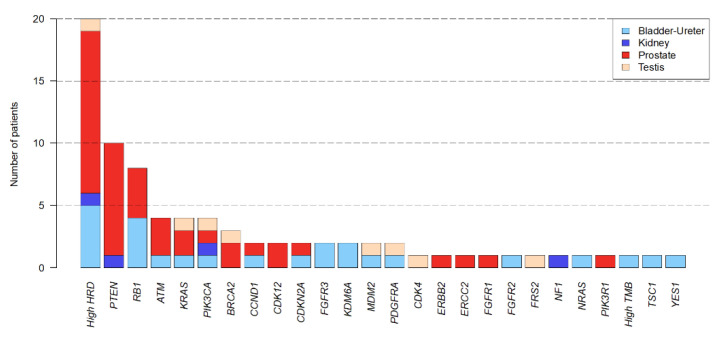
List and incidence of AGAs. The 26 genes and genomic scores identified as AGAs are ordered from left to right by decreasing number of samples with alterations. For each AGA, the number of patients with AGA per cancer type is color-coded according to the legend.

Seventy-nine AGAs were identified in real-time; 38 patients had at least one AGA (59%) with a median number per patient of 2 (range, 1–5). AGAs included 58 single-gene alterations (32 mutations, 26 CNAs) concerning 24 genes, and 21 global genomic scores (20 high HRD, 1 high TMB) (Figure 3). High HRD was the most frequent AGA (*N =* 20 patients), followed by alterations of PTEN (*N =* 10), RB1 (*N =* 8), ATM, PIK3CA, KRAS (*N =* 4), and BRCA2 (*N =* 3). Single-gene AGAs altered the PIK3/AKT/MTOR (*N =* 16 patients), cell cycle (*N =* 12), DNA repair (*N =* 10), tyrosine kinase receptors (*N =* 9), and RAS/MAPK (*N =* 6) pathways.

**Figure 4:**
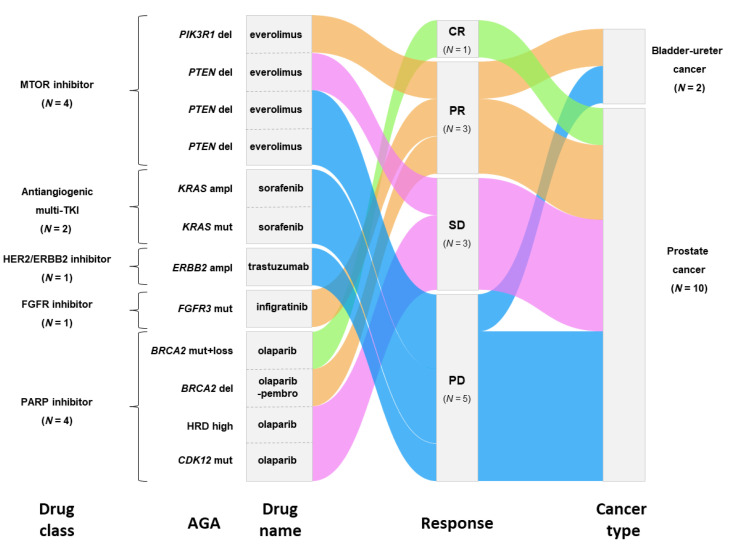
Matched therapies, corresponding AGAs, and therapeutic responses. Matched therapies (drug class and drug name) and corresponding targeted AGAs are indicated. Objective responses are displayed as alluvial plots linking drug, response, and cancer type: responses ordered from CR to PD. CR = complete response, PR = partial response; SD = stable disease, and PD = progressive disease.

Among the 38 patients with AGA, 12 received a matched therapy as new therapy for progression after delivery of the molecular report (therapy 2), representing 19% (95%CI 10–30) of the enrolled patients. These 12 patients had received a median of 3.5 (range, 1–9) prior lines of systemic therapy for advanced disease (Table 1). AGAs leading to matched therapy were PTEN deletion (*N =* 3), BRCA2 alterations (*N =* 2), KRAS mutation/amplification (*N =* 2), and FGFR3 mutation, ERBB2 amplification, PIK3R1 deletion, CDK12 mutation, and high HRD score (*N =* 1) (Figure 4). The corresponding matched therapies were given as single-agent (one patient received olaparib-pembrolizumab combination), the most frequent ones being MTOR inhibitors (*N =* 4) and PARP inhibitors (*N =* 4), followed by kinase inhibitors (Figure 4). Eighty-three percent of 12 patients (83%) were treated within phase I/II trials.

The reasons for not giving a matched therapy to the 26 other patients with AGA were (Figure 1): lost to follow-up (*N =* 4), palliative care (*N =* 10), delivery of a non-matched therapy (*N =* 12) for lack of available clinical trial (*N =* 6), patient not enrollable (*N =* 3), therapy already received during the previous lines (*N =* 2), or physicians’ choice (*N =* 1).

### 3.4. Outcome of Patients Treated after Delivery of the Molecular Report

Analysis concerned the 37 patients treated with a systemic therapy for progression after delivery of the molecular report (therapy 2). Among the 64 enrolled patients (Figure 1), 21 had not been treated (palliative care for 19, death before the genomic results for 2), and 6 were lost to follow-up. The 37 treated patients included 9 with a non-exploitable molecular profile, 4 without any AGA identified, and 24 with an identified AGA (12 receiving a matched therapy, 12 a non-matched therapy). We compared the outcome of patients treated with matched therapy (“matched therapy group”; *N =* 12) with that of patients treated with non-matched therapy (“non-matched therapy group”; *N =* 25). The characteristics of both groups are shown in Table 1. There was no significant difference, except for a younger age in the “non-matched therapy group” (*p* = 3.20 × 10^−2^). Therapy 2 in the “non-matched therapy group” (Table S3) was chemotherapy (*N =* 13), targeted therapy (*N =* 6), hormone therapy (*N =* 4), and immune therapy (*N =* 2).

**Figure 5:**
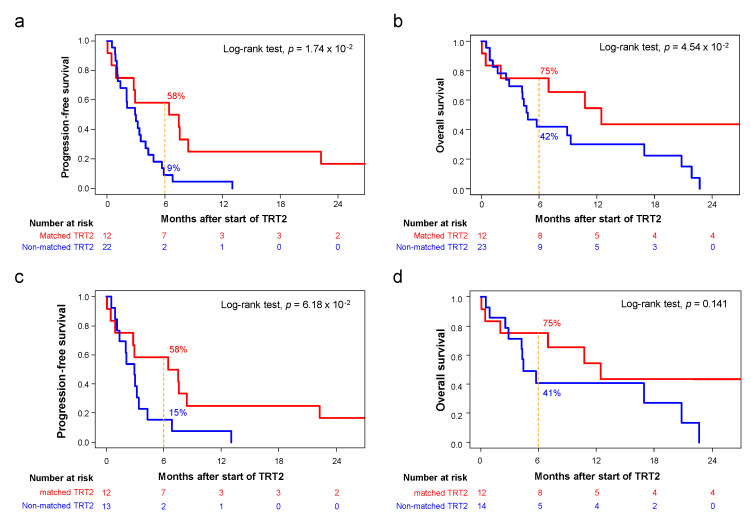
Clinical outcome in patients treated with “matched therapy” versus “non-matched therapy”. (**a**) Kaplan–Meier curve of PFS2 in patients treated with “matched therapy” (red curve) and in patients treated with “non-matched therapy” (blue curve). (**b**) Similar to a, but for OS. (**c**) Similar to a but limited to patients with PC or bladder/ureter cancer. (**d**) Similar to c, but for OS.

In term of efficacy, 5/12 patients (42%) in the “matched therapy group” had a PFS2/PFS1 ratio ≥ 1.3 versus only 1/25 (5%) in the “non-matched therapy group”. In univariate analysis for PFS2/PFS1 ratio ≥ 1.3, the type of therapy (matched versus non-matched) was the only significant variable (*p* = 1.36 × 10^−2^; Table S4). The 6-month PFS2 was longer in the “matched-therapy group” (58%, 95%CI 36–94) than in the “non-matched therapy group” (9%, 95%CI 2-34; *p* = 1.74 × 10^−2^; Figure 5a). In term of RECIST response, in the “matched therapy group” (Figure 4), one BRCA2-mutated PC patient (8%) experimented complete response (CR: 8%) with olaparib, three patients displayed partial response (PR: 25%), including one with urothelial cancer and FGFR3 mutation treated with infigratinib, one with PC and BRCA2 deletion treated with pembrolizumab/olaparib combination, and one with PC and PIK3R1 deletion treated with everolimus. Three patients presented stable disease (SD: 25%) and five were progressive (PD: 42%). The objective response (OR) rate was 33% (95%CI 11–65) and the disease control (DC) rate (CR + PR + SD) was 58% (95%CI 28-85). In the “non-matched therapy group”, these rates were 13% (95%CI 3–35) and 22% (95%CI 7–44), respectively. The comparison between both groups showed a trend towards better DC rate in the “matched therapy group” (*p* = 5.9 × 10^−2^). The 6-month OS was longer in the “matched-therapy group” (75%, 95%CI 54–100) than the “non-matched therapy group” (42%, 95%CI 26–69; *p* = 4.54 × 10^−2^; Figure 5b). Because all patients in the “matched therapy group” had a PC or a bladder/ureter cancer (*N* = 12), we repeated the same analysis by focusing on patients with PC or bladder/ureter cancer only in the “non-matched therapy group” (*N* = 15). Here too, the percentage of patients with a PFS2/PFS1 ratio ≥ 1.3 was higher in the “matched therapy group” (42%) than in the “non-matched therapy group” (7%; *p* = 0.065). The 6-month PFS2 was 58% (95%CI 36–94) in the “matched-therapy group” *versus* 15% (95%CI 4–55) in the “non-matched therapy group”; *p* = 6.18 × 10^−2^; Figure 5c), and the corresponding 6-month OS were 75% (95%CI 54–100) and 41% (95%CI 21–78), respectively (*p* = 0.141; Figure 5d).

## 4. Discussion

Fifty-nine percent of AUC patients enrolled in PERMED-01 presented at least one AGA and 19% received an AGA-matched therapy, which provided a PFS2/PFS1 ratio ≥ 1.3 in 42% of cases (8% of enrolled patients). For comparison, a ratio ≥ 1.3 was observed in 5% of patients treated with a non-matched therapy.

Compared to other precision medicine trials, we included two design modifications: analysis of the largest gene panel tested by tNGS and extended to clinically relevant genomic scores, and profiling of new biopsies done inside the trial. The use of a new biopsy rather than archival samples was justified by the known temporal evolution of the tumor genome with emergence of subclonal alterations [18]. Feasibility was good with 77% of patients displaying an exploitable profile validated within a median time compatible with clinical use (53 days). Most of genomics failures were due to insufficient quantity and/or quality of biopsy. The safety of biopsy was good with only one patient displaying a grade 3 fever after prostate biopsy, with complete recovery after antibiotic treatment.

We found somatic alterations consistent with the literature, such as AR, TP53, FOXA1, SPOP, PTEN, or ATM alterations in PC [23], and TP53, KDM6A, RB1, ATM, FAT1, or ARID1A alterations in bladder urothelial cancer [7]. As expected, the two PCs with bi-allelic CDK12 alteration showed a focal tandem duplication profile in aCGH. CDK12 promotes DNA repair through the regulation of homologous recombination genes, with a suggestion that CDK12 bi-allelic inactivation was associated with PARP-inhibitor sensitivity [24]. One of the two patients was treated with olaparib and showed disease stabilization over 8 months. Twenty-four genes displayed germline variants, including four PGVs (15% of tested patients) and concerning DNA repair genes. One patient had been referred for oncogenetics consultation. The 15% rate is close to the 10% reported in a subcohort of 127 patients with AUC [25]. Whether this relatively high rate of PGVs identification should lead to systematic oncogenetics consultation and germline sequencing in patients with AUC deserves investigation.

We identified AGAs in real-time in 59% of enrolled patients, a proportion inferior to that reported in the whole PERMED-01 population (71%), which included many different cancer types [16]. For comparison, we identified in the literature 10 precision medicine studies that included AUC patients and were informative regarding this point: seven were dedicated to all cancer types, including urological cancers [11,26,27,28,29,30,31], and three were dedicated to a specific urological cancer type, including both molecular screening and delivery of the therapy matched to pre-defined AGAs [5,14,15]. Through these 10 studies, the median percentage of patients with AUC identified with AGA was 28% (10–48%). For example, in the first category, it was 40% in the ProfiLER trial based on t-NGS and aCGH [30], and 42% in the Dutch study based on WGS [31]. In the second category, the PROfound study focused on deleterious or suspected deleterious alterations in 15 genes selected for their role in homologous recombination in PC patients [14]. A qualifying alteration in one or more of these genes was detected in 28% of 2792 patients, a percentage close to the present cohort when analysis was limited to the same genes (34%). However, comparison with the literature is difficult because the gene panels and techniques used are different; for example, the number of “candidate genes” tested by tNGS was smaller across the 10 previous studies (median 50 genes (range, 9–410)) than in our series (≥494 in 92% of our exploitable profiles). Even more critical, AGA definitions are different. The use of scales for definition and level of actionability of molecular alterations, such as ESCAT or OncoKB, which we used here [21], and their regular updating will help in the future such comparisons.

On progression after enrollment, 19% of enrolled patients received a matched therapy, representing 32% of patients with AGA, a proportion similar to that reported in the whole PERMED-01 population (17%) [16]. These results are close to the 12% (4–29%) of enrolled patients (and 42% of patients with AGA) found in nine informative published studies [5,11,14,26,27,28,30,32,33]. This low rate of patients treated with matched therapy has several explanations. The main one is the advanced and previously multi-treated status of patients and poor general status, which led to palliative care or loss to follow-up. Among treated patients, the main difficulty was the access to the matched therapy because of lack of clinical trials, and the non-enrollment in available trials mainly because of performance status. Recently, clinical trials were designed to facilitate access to targeted matched therapies such as PROfound in PC or Biscay in bladder cancer [5,14,15], the two cancers treated with matched therapy in our series. In these studies, drug administration was planned from the start of the study for patients presenting a relevant AGA. In the future, inclusion of patients earlier in the disease course will decrease the risk of clinical deterioration before the delivery of AGA-matched therapy. Such an approach is being tested in trials such as SAFIR02-breast and -lung (NCT02117167, NCT02299999) or MULTISARC (NCT03784014). In parallel, improving the number of and accessibility to clinical trials of matched therapies, alone and in combination, should improve this point, thanks to less restrictive patients’ eligibility criteria [34] and wider selection of participating centers in phase I/II trials. Indeed, it seems that the highest efficacy of precision medicine is observed in patients treated in large academic centers with broad phase I/II trials portfolio [11,26,29,32,33,35].

PERMED-01 was not primarily designed to assess the clinical efficacy of AGA-matched therapy. However, our results suggest a clinical benefit in patients with AUC. Forty-two percent of patients (42%) in the “matched therapy group” had a PFS2/PFS1 ratio ≥1.3 versus 5% in the “non-matched therapy group”. This percentage is close to that observed in the whole PERMED-01 (37%) [16] and MOSCATO (33%) [33] series. The OR and DC rates were higher in the “matched therapy group” (33% and 58% respectively) than the “non-matched therapy group” (13% and 22%), as was the 6-month OS (75% vs. 42%). In the literature, few “all cancer type studies” present separately the efficacy results for patients with AUC. In the IMPACT trial, no objective response was observed in the four patients treated with matched therapy [28]. In the PREDICT–UCSD trial, the disease control rate was similar in the 22 matched-treated patients (23%) and the 158 non-matched treated patients (25.5%) [32]. In ProfiLER, no objective response was observed in three patients treated with matched therapy [30]. In MOSCATO, among the 29 AUC patients treated with matched therapy, 42% showed a PFS2/PFS1 ratio ≥ 1.3 [33], a rate similar to ours. Among the patients treated with “matched therapy” in our series, the most frequently delivered matched therapies were everolimus and olaparib (4 patients each), followed by sorafenib (2 patients). Our results showed higher efficacy in term of tumor response with olaparib than with everolimus and sorafenib. Recently, the PROfound trial found that olaparib was somewhat effective in cases of advanced PC with HRD (BRCA1, BRCA2 or ATM) [14], as observed with our two patients with BRCA2 inactivation and partial and complete response to olaparib. Lesser efficacy of everolimus and sorafenib, as compared to olaparib, may suggest that these drugs are not as effective for the indicated lesions and/or that the AGA is not yet explored well enough to exploit in precision medicine. However, the numbers of patients are very small and of course, the analysis is considerably biased and cannot serve to establish the value of the research strategy. These results call for further confirmation studies and only randomized trials comparing “AGA-matched therapy” *versus* conventional care will need to be implemented.

However, our percentage suggests benefit in only 8% of enrolled patients, a rate classically reported in other precision medicine studies [9]. Of course, the small number of patients and the design of these studies, including ours, preclude any definitive conclusion. This relatively limited benefit of precision medicine trials reported to date results from different reasons: (i) enrollment of end-staged metastatic patients heavily pre-treated with poor performance status, showing rapid disease progression not compatible with the time to get the results, thus leading to a significant drop-off between the MTB recommendations and the initiation of matched therapy; (ii) various tumor and pathological types, introducing a source of variability into the analysis that bias the results, notably because the predictive impact of a molecular alteration depends on the cancer type; (iii) clonal heterogeneity of metastatic cancers highly mutated with plastic genomes allowing adaptation and resistance to treatments; (iv) limited functional relevance of AGA due to the absence of reliable tests; (v) limitation of analyses to DNA CNA and mutations, although analyses of mRNA and protein expression and the search for actionable fusion genes and signatures of pathway alteration should be considered; and (vi) limited access to clinical trials of appropriate matched therapies, with restrictive patients’ eligibility criteria [34] and limited selection of participating centers in phase I/II trials of innovative drugs. The size of gene panel to be tested using NGS remains controversial (tNGS versus WES and/or WGS) and clinical trials are testing this issue (NCT03163732). Inclusion of patients earlier in the disease course (less complex genomic profile and lesser risk of clinical deterioration), and with unique cancer type showed interesting results in PC [14] and bladder cancer [5], and more recently in clear-cell renal cell carcinoma [36].

## 5. Conclusions

A prospective extensive molecular profiling of AUC based on a new tumor biopsy allowed identification of AGAs in 59% of patients, delivery of a matched therapy on progression in 19% (mainly PC), and observation of a clinical benefit (PFS2/PFS1 ratio ≥ 1.3) in 8%. This rather limited benefit has several explanations common to many precision medicine trials, the design of which must be improved at all levels (patients’ selection, new biopsy, integrated analyses, AGAs definition/relevance, access to matched therapies). However, even if our results about efficacy are significant, we acknowledge that our study displays limitations, including the small number of patients in the compared groups with 12 patients in the “matched therapy group”, and 25 in the “non-matched therapy group”, and the imbalance of characteristics between these groups with a strong trend for more patients with PC in the first one (83%) than in the second one (44%). Yet, a pooled analysis of patients with AUC treated in other similar precision medicine trials (ProfiLER, MOSCATO…) [26,27,28,29,30,31,32,33] might be a solution to improve these limitations. To date, such an approach remains limited to the research field, and randomized trials are warranted for an effective future assessment of the clinical feasibility and (eventual) benefit of this multimodal therapy approach.

## Figures and Tables

**Table 1:** Patients’ characteristics at inclusion.

Characteristics		All Patients (*N =* 64)	Matched TRT2 (*N =* 12)	Non-Matched TRT2 (*N =* 25)	*p*-Value *
**Age, years**					
	Median (range)	65.55 (28–83)	70.5 (55–83)	62.52 (28–81)	3.2 × 10^−2^
**Sex**					0.168
	Male	58 (91%)	12 (100%)	19 (76%)	
	Female	6 (9%)	0 (0%)	6 (24%)	
**ECOG performance status**					0.890
	0	34 (57%)	7 (58%)	14 (61%)	
	1	23 (38%)	4 (33%)	8 (35%)	
	2	3 (5%)	1 (8%)	1 (4%)	
**Cancer type**					0.071
	Prostate	39 (61%)	10 (83%)	11 (44%)	
	Bladder-Ureter	12 (19%)	2 (17%)	4 (16%)	
	Kidney	9 (14%)	0 (0%)	7 (28%)	
	Testicular	4 (6%)	0 (0%)	3 (12%)	
**Site of the biopsy**					0.493
	Liver	22 (36%)	4 (33%)	7 (32%)	
	Lymph node	13 (21%)	3 (25%)	4 (18%)	
	Lung	7 (11%)	0 (0%)	5 (23%)	
	Peritoneum	2 (3%)	0 (0%)	1 (5%)	
	Prostate	10 (16%)	3 (25%)	3 (14%)	
	Other	7 (11%)	2 (17%)	2 (9%)	
**Pathological type**					0.341
	Carcinoma	59 (92%)	12 (100%)	21 (84%)	
	Germ cell tumor	3 (5%)	0 (0%)	2 (8%)	
	Other	2 (3%)	0 (0%)	2 (8%)	
**Extension stage**					1
	Locally advanced	1 (2%)	0 (0%)	1 (4%)	
	Metastatic	63 (98%)	12 (100%)	24 (96%)	
**Number of metastatic sites**					
	Median (range)	2.44 (0–4)	2.17 (1–4)	2.48 (0–4)	0.433
**Number of previous treatment lines for advanced disease**					
	Median (range)	3.50 (0–11)	3.50 (1–9)	3.0 (0–8)	0.229

*, *p*-value for the matched vs. non-matched TRT2 comparison.

## Data Availability

The dataset supporting the conclusions of this article has been deposited in the European Genome-phenome Archive (EGA) repository: accession EGAS00001004554 and https://www.ebi.ac.uk/ega/home for t-NGS data (https://ega-archive.org/studies/EGAS00001004554, accessed on 8 June 2021) and in the ArrayExpress database at EMBL-EBI under the E-MTAB-9998 accession number for array-CGH data (https://www.ebi.ac.uk/arrayexpress/experiments/E-MTAB-9998/, accessed on 19 January 2021).

## Supplementary Materials

The following supporting information can be downloaded at: https://www.mdpi.com/article/10.3390/cancers14092275/s1, Figure S1: Repertoire of somatic alterations in prostate cancers; Figure S2: Violin plots of HRD scores in urological cancers according to the presence/absence of mono- and bi-allelic pathogenic alterations of genes involved in homologous recombination; Table S1: Gene panels used in the t-NGS analysis; Table S2: Pathogenic germline variants identified; Table S3: Systemic therapies 2 received by patients in the “matched therapy” and “non-matched therapy” groups; Table S4: Univariate analysis for PFS2/PFS1 ratio in patients treated for disease progression after enrollment.
