# Supplementary material for: Molecular Profiles of Advanced Urological Cancers in the PERMED-01 Precision Medicine Clinical Trial
Source: Cancers (Basel). 2022 May 3;14(9):2275. doi: 10.3390/cancers14092275 (PMC9100924; doi:10.3390/cancers14092275)
Supplement: Supplementary file 1 [file cancers-14-02275-s001.zip › Figure.pdf]

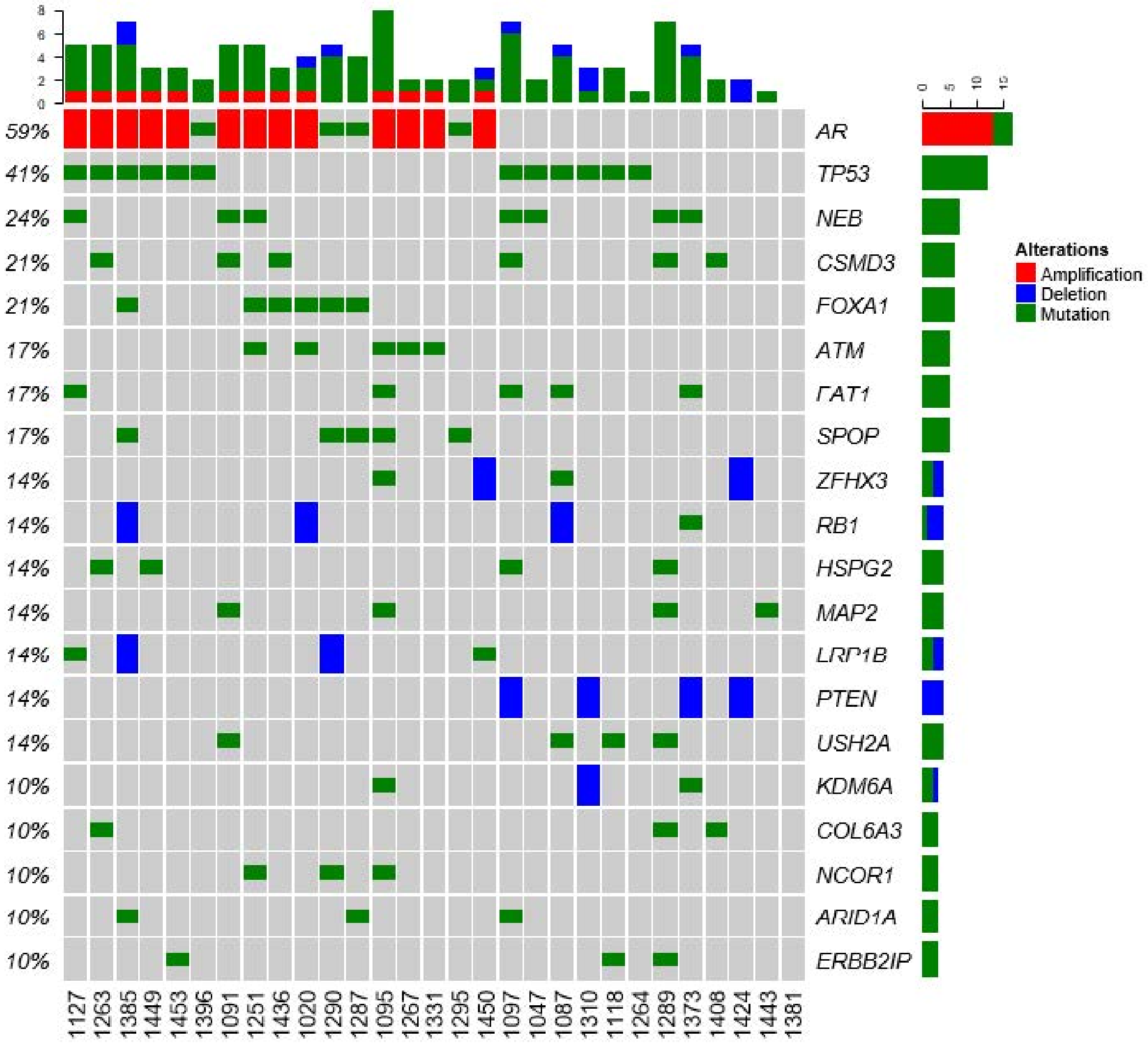

Figure S1. Repertoire of somatic alterations in prostate cancers.

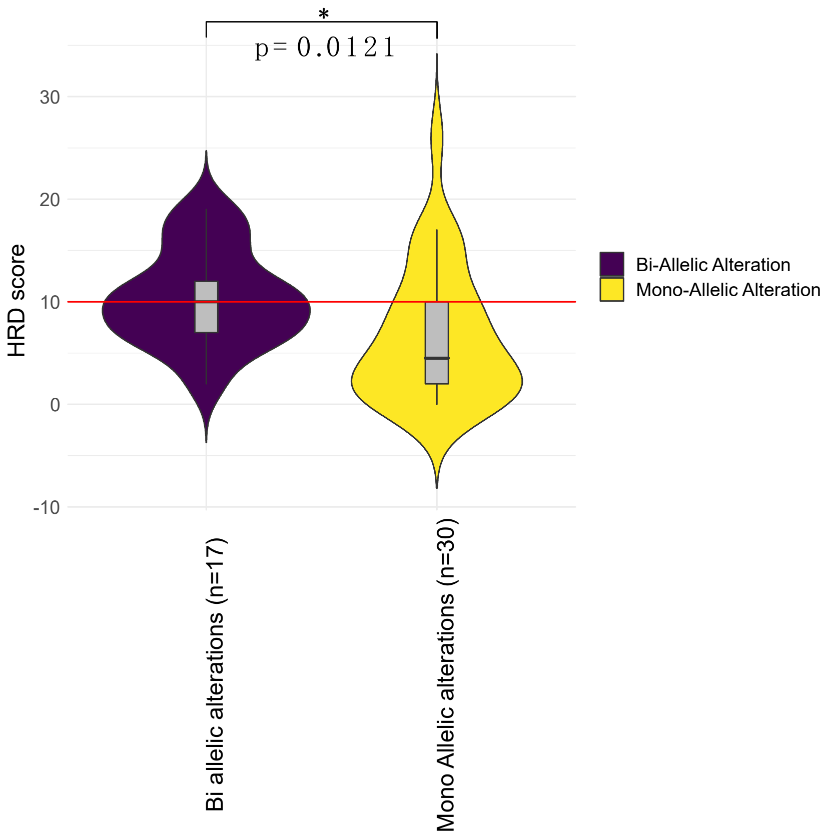

Figure S2. Violin plots of HRD scores in urological cancers according to the presence/absence of mono- and bi-allelic pathogenic alterations of genes involved in homologous recombination.
